# Numerical simulation for bioconvectional flow of burger nanofluid with effects of activation energy and exponential heat source/sink over an inclined wall under the swimming microorganisms

**DOI:** 10.1038/s41598-021-93748-x

**Published:** 2021-07-12

**Authors:** Hassan Waqas, Umar Farooq, Aqsa Ibrahim, M. Kamran Alam, Zahir Shah, Poom Kumam

**Affiliations:** 1 Department of Mathematics, Government College University Faisalabad, Layyah Campus, Faisalabad, 31200 Pakistan; 2 Department of Physics, Government College University Faisalabad, Faisalabad, 38000 Pakistan; 3 Department of Pure & Applied Mathematics, The University of Haripur, Khyber Pakhtunkhwa, 22620 Pakistan; 4 Department of Mathematical Sciences, University of Lakki Marwat, Lakki Marwat, 28420 Khyber Pakhtunkhwa Pakistan; 5 Center of Excellence in Theoretical and Computational Science (TaCS-CoE), Faculty of Science, Thonburi (KMUTT), King Mongkut’s University of Technology, 126 Pracha Uthit Rd., Bang Mod, Thung Khru, Bangkok, 10140 Thailand; 6 Fixed Point Research Laboratory, Fixed Point Theory and Applications Research Group, Center of Excellence in Theoretical and Computational Science (TaCS-CoE), Faculty of Science, King Mongkut’s University of Technology Thonburi (KMUTT), 126 Pracha Uthit Rd., Bang Mod, Thung Khru, Bangkok, 10140 Thailand; 7 Department of Medical Research, China Medical University Hospital, China Medical University, Taichung, 40402 Taiwan

**Keywords:** Engineering, Mathematics and computing, Physics

## Abstract

Nanofluids has broad applications such as emulsions, nuclear fuel slurries, molten plastics, extrusion of polymeric fluids, food stuffs, personal care products, shampoos, pharmaceutical industries, soaps, condensed milk, molten plastics. A nanofluid is a combination of a normal liquid component and tiny-solid particles, in which the nanomaterials are immersed in the liquid. The dispersion of solid particles into yet another host fluid will extremely increase the heat capacity of the nanoliquid, and an increase of heat efficiency can play a significant role in boosting the rate of heat transfer of the host liquid. The current article discloses the impact of Arrhenius activation energy in the bioconvective flow of Burger nanofluid by an inclined wall. The heat transfer mechanism of Burger nanofluid is analyzed through the nonlinear thermal radiation effect. The Brownian dispersion and thermophoresis diffusions effects are also scrutinized. A system of partial differential equations are converted into ordinary differential equation ODEs by using similarity transformation. The multi order ordinary differential equations are reduced to first order differential equations by applying well known shooting algorithm then numerical results of ordinary equations are computed with the help of bvp4c built-in function Matlab. Trends with significant parameters via the flow of fluid, thermal, and solutal fields of species and the area of microorganisms are controlled. The numerical results for the current analysis are seen in the tables. The temperature distribution increases by rising the temperature ratio parameter while diminishes for a higher magnitude of Prandtl number. Furthermore temperature-dependent heat source parameter increases the temperature of fluid. Concentration of nanoparticles is an decreasing function of Lewis number. The microorganisms profile decay by an augmentation in the approximation of both parameter Peclet number and bioconvection Lewis number.

## Introduction

Due to the significant applications in the engineering field, nanofluids have drawn the interest of many scientists. The heat transition of convection liquids such as ethylene glycol, kerosene, water and oil can be used in a wide variety of engineering tools, such as electron and heat transfer instruments. Nanofluids are the combination of smaller nanomaterials and a base fluid. As a consequence, the presence of micro solid objects in typical fluids has enhanced the characteristics of heat transformation. There are many potentials uses of nanofluids for heat transfer, namely cooling systems, air conditioners, chillers, microelectronics, computer microchips, diesel engine oil and fuel cells. It should be remembered that the thermal conductivity of nanomaterials is improved by volume fraction, particulate size, pressure, including thermal conductivity. Nanotechnology is of considerable interest in a variety of industries, including chemical and metallurgical equipment, shipping, macroscopic artifacts, medical treatments, and electricity generation. Nanofluids are mixtures of nanometer-sized particulate suspensions with conventional fluids, including one that was presented by Choi^1^. Buongiorno^2^ explores the two peculiar sliding mechanisms, in particular, the Brownian diffusion and thermophoresis influence, to enhance the normal convection rate of the heat energy distribution. Venkatadri et al.^3^ researched a melting heat transport of an electrical nanofluid flow conductor towards an exponentially shrank/extended porous layer with nonlinear radiative Cattaneo-Christov heat flux under a magnetic field. Mondal et al.^4^ have studied the impact of the heat exchange of magnetohydrodynamics on the stagnation point flow over the extended or decreasing surface by homogeneous chemical reactions. Ying et al.^5^ examined radiative heat transmission of molten salt-based flow of nanofluid over a non-uniform heat flux. Zainal et al.^6^ tracked the MHD hybrid nanofluid flow to the porous expansion/reduction sheet at the presence of a quadratic momentum. Eid et al.^7^ identified a shift in thermal conductivity, namely heat transfer effects on the magneto-water nanofluid flow in a porous slippery channel. Numerous researchers are involved in the Burger nanofluid seen in Ref^8–23^.

The process of bioconvection can be described as the swimming up of microbes in materials, which are less dense than water. owing to the advanced concentration of microorganisms, above that the layer of substances happens to too thick and delicate, which allows the microorganisms to break down owing to the bioconvection flow. Microorganisms, many of which are older organisms on the globe known as human beings, are very important in many ways. It is defined as a type of growth of microorganism substances, such as bacteria or algae, due to the up-swimming microorganism. Bioconvection has many uses in the world of biochemistry and bioinformatics. The Bioconvection process is used by bioengineering in diesel fuel goods, bioreactors, and fuel cell engineering. Platt^24^ was the very first person to describe bioconvection phenomena. Unstable density distributions were adopted as a technique for the arrangement of suspensions of swim motile microorganisms and the term bioconvection was created. Kuznetsov^25^ subsequently introduced this idea based on nanofluids, namely gyrotactic motile microorganisms, suggesting that the resultant large-scale flow of fluid produced by self-propelled motile gyrotactic microorganisms increases the mixture and prevents nanomaterials aggregation in nanofluids. Haq et al.^26^ studied the flow properties of Cross Nanoparticles across expanded surfaces subject to Arrhenius activation energy and magnetization field. Ahmad et al.^27^ examined a bioconvection nanofluid flow comprising gyrotactic motile microorganisms with a chemical reaction allowance through a porous medium past a stretched surface. Elanchezhian et al.^28^ worked on the rate of motile gyrotactic microorganisms in the bioconvective nanofluid flow of Oldroyd-B past a stretching sheet with a mixing convective and inclination magnetization area. Bhatti et al.^29^ performed a mathematical analysis on the migration of motile swimming microorganisms in non-Newtonian blood-based nanoliquid by anisotropic artery restriction. Khan et al.^30^ illustrated the essential rheological characteristics of Jeffrey's gyrotactic motile microorganism–like nanofluid by rapid development. Shafiq et al.^31^ assessed the rate of heat and mass transition of gyrotactic microorganisms with the second-grade nanofluid flow. Kotnurkar et al.^32^ addressed the bioconvection of 3rd-grade nanoliquid flowing by copper-blood nanofluids in porous walls, consisting of motile species. Muhammad et al.^33^ recognized the time-dependent motion of thermophysical magnetization Carreau nanofluids, which convey motile microorganisms via a spinning wedge through velocity slip as well as thermal radiation features. Farooq et al.^34^ have introduced an entropic example of the 3-D bioconvective movement of nanoliquid across a linearly spinning plate in the absence of magnetic influences. Hosseinzadeh et al.^35^ investigated the flow of motile microorganisms and nanotechnology through a 3-D stretching cylinder. Any important and most recent work of bioconvection swimming fluid microorganisms has been analyzed analytically by a variety of fascinating investigators^36–40^.

Our inspiration of the present study is to examine the model of the Burger nanofluid with activation energy and exponential heat source/sink through inclined wall. The behaviors of Brownian motion and thermophoresis diffusion effects are scrutinized. Bioconvection and motile microorganisms is also discussed. The novelty of this work is investigating the 2D flow of Burger nanoliquid past an inclined wall. The dimensionless ODEs are tackled with shooting method to reduce the order via bvp4c MATLAB tool. The careful study of literature shows that the mathematical formulation established in this communication is novel and has not been talked before as per the author’s data. The physical performance of parameters via flow profiles are survey via graphical and tabular data.

## Mathematical formulation

**Figure 1:**
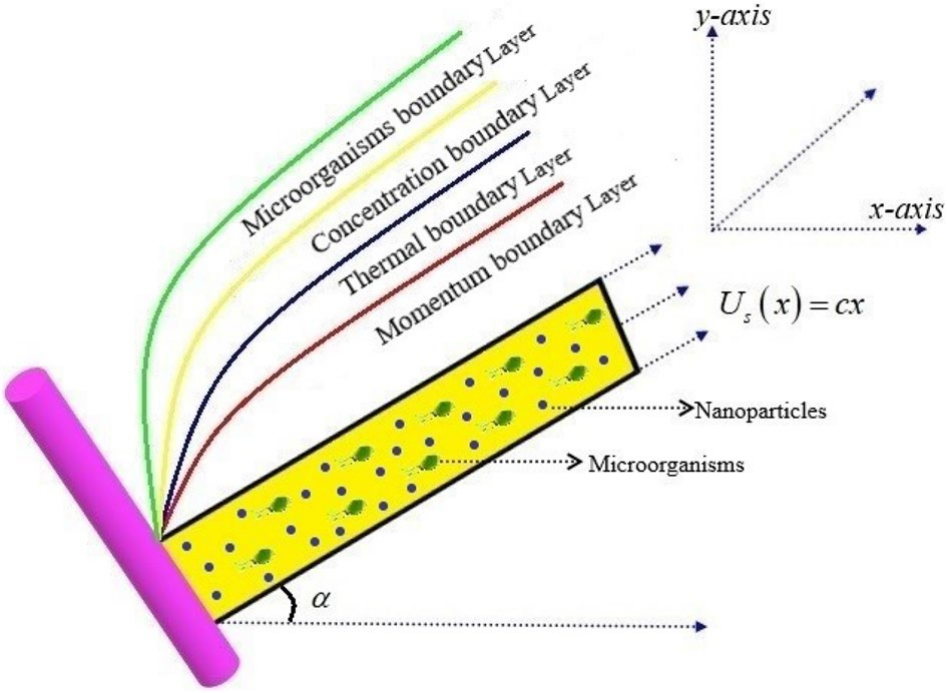
Flow pattern of the problem.

This model appraises the two-dimensional Bioconvectional flow of Burgers nanofluid containing swimming gyrotactic microorganisms over a vertical inclined wall. The Brownian motion and thermophoresis diffusion are considered for nanofluid. Heat and mass transfer aspects are found to be associated with the exponential space based heat source. The velocity of the wall is $U_{s} \left( x \right) = cx$ and the magnetic field is along the transverse direction. The inclined wall is clarified in Fig. 1. Basic laws describing the conservation of mass and momentum yield.

1 $$div{\mathbf{V}} = 0,$$

2 $$\rho \frac{{D{\mathbf{V}}}}{{Dt}} = - \nabla p + div{\mathbf{S}},$$

The extra stress tensor of burger fluid model is

3 $${\mathbf{S}} + \lambda _{1} \frac{{D{\mathbf{S}}}}{{Dt}} + \lambda _{2} \frac{{D^{2} {\mathbf{S}}}}{{Dt^{2} }} = \mu \left( {{\mathbf{A}}_{1} + \lambda _{3} \frac{{D{\mathbf{A}}_{1} }}{{Dt}}} \right).$$

The modeled boundary layer equations for the nanofluid flow model are represented as follows^41^: In the expirations $\left( {\lambda _{3} } \right)$ is the retardation effect and $\left( {\lambda _{1} \& \lambda _{2} } \right)$ are relaxation effects. It is marked out here that the outcomes for the Oldroyd-B fluid model can be deduced for $\left( {\lambda _{2} = 0} \right)$ and the findings for the Maxwell fluid model can be reduced $\left( {\lambda _{2} = \lambda _{3} = 0} \right)$. Also, the effects of the Fluid model can be extracted by specifying $\left( {\lambda _{1} = \lambda _{2} = \lambda _{3} = 0} \right)$.

4 $$\begin{gathered} uu_{x} + vu_{y} + \lambda _{2} \left( {u^{2} u_{{xx}} + v^{2} u_{{yy}} + 2uvu_{{xy}} } \right) + \lambda _{3} \left( \begin{gathered} u^{3} u_{{xxx}} + v^{3} u_{{yyy}} + u^{2} \left( {u_{{xx}} u_{x} - u_{y} v_{{xx}} + 2v_{x} u_{{xy}} } \right) \hfill \\ 3v^{2} \left( {v_{y} u_{{yy}} + u_{y} u_{{xy}} } \right) + 3uv\left( {uu_{{xxy}} + vu_{{xyy}} } \right) \hfill \\ 2uv\left( {u_{y} u_{{xx}} + v_{x} u_{{yy}} + v_{y} u_{{xy}} - u_{y} v_{{xy}} } \right) \hfill \\ \end{gathered} \right) \hfill \\ = \nu \left\{ {u_{{yy}} + \lambda _{3} \left( {uu_{{xyy}} + vu_{{yyy}} - u_{x} u_{{yy}} - u_{y} v_{{yy}} } \right)} \right\} - \frac{{\sigma B_{0}^{2} }}{{\rho _{f} }}\left( {u + \lambda _{1} vu_{y} + \lambda _{2} \left\{ \begin{gathered} uv_{x} u_{y} - vu_{x} u_{y} \hfill \\ + uvu_{{xy}} + v^{2} u_{{yy}} \hfill \\ \end{gathered} \right\}} \right) \hfill \\ + \cos \alpha \frac{1}{{\rho _{f} }}\left[ \begin{gathered} \left( {1 - \Phi _{f} } \right)\rho _{f} \beta ^{{**}} g*\left( {T - T_{\infty } } \right) - \left( {\rho _{p} - \rho _{f} } \right)g^{*} \left( {\Phi - \Phi _{\infty } } \right) \hfill \\ - \left( {N - N_{\infty } } \right)g^{*} \gamma \left( {\rho _{m} - \rho _{f} } \right) \hfill \\ \end{gathered} \right], \hfill \\ \end{gathered}$$

5 $$\begin{gathered} uT_{x} + vT_{y} = \alpha _{m} T_{{yy}} + \tau \left\{ {D_{B} \Phi _{y} T_{y} + \frac{{D_{T} }}{{T_{\infty } }}\left( {T_{y} } \right)^{2} } \right\} + \left( {\frac{{16\sigma _{q} T_{\infty }^{3} }}{{3k^{*} \left( {\rho c_{p} } \right)_{f} }}} \right)T_{{yy}} \hfill \\ + \frac{{Q_{T}^{*} }}{{\left( {\rho c_{p} } \right)_{f} }}\left( {T - T_{\infty } } \right) + \frac{{Q_{E}^{*} }}{{\left( {\rho c_{p} } \right)_{f} }}\left( {T - T_{\infty } } \right)\exp \left( { - \left( {\frac{c}{{v_{f} }}} \right)^{{0.5}} ny} \right), \hfill \\ \end{gathered}$$

6 $$u\Phi _{x} + v\Phi _{y} = D_{B} \Phi _{{yy}} + \frac{{D_{T} }}{{T_{\infty } }}T_{{yy}} - Kr^{2} \left( {\Phi - \Phi _{\infty } } \right)\left( {\frac{T}{{T_{\infty } }}} \right)^{m} \exp \left( {\frac{{ - E_{a} }}{{K_{1} T}}} \right),$$

7 $$uN_{x} + vN_{y} = D_{m} \left( {N_{{yy}} } \right) - \frac{{bW_{c} }}{{\left( {\Phi _{s} - \Phi _{\infty } } \right)}}\left[ {\partial _{y} \left( {N\Phi _{y} } \right)} \right],$$

With relative boundary conditions^42^:

8 $$\left. \begin{gathered} u = U_{s} ,\left. {\left. {\mu u_{y} } \right|_{{y = 0}} \sigma _{x} } \right|_{{y = 0}} = \left. {\sigma _{T} T_{x} } \right|_{{y = 0}} \left. { - \sigma _{\Phi } \Phi _{x} } \right|_{{y = 0}} , \hfill \\ - kT_{y} = h_{f} \left( {T_{s} - T} \right), - D_{B} \Phi _{y} = h_{g} \left( {\Phi _{s} - \Phi } \right), \hfill \\ - D_{m} N_{y} = h_{n} \left( {N_{s} - N} \right),\,at\,y = 0, \hfill \\ u = 0,v = 0,T \to T_{\infty } ,\Phi \to \Phi _{\infty } ,N \to N_{\infty } \,as\,y \to \infty . \hfill \\ \end{gathered} \right\},$$

Here in the above equation $\left( {u\& v} \right)$ are velocity components, $\left( {\alpha _{m} } \right)$ is thermal diffusivity, $\left( \alpha \right)$ is the inclination of the wall, $\left( {\rho _{f} } \right)$ is density, $\left( {g^{*} } \right)$ acceleration due to gravity, $\left( \sigma \right)$ is electric conductivity, $\left( T \right)$ is temperature, $\sigma _{q}$ signifies Boltzmann constant, $k^{*}$ denotes means absorption coefficient, $\left( {D_{B} } \right)$ is Brownian motion coefficient, $\left( {\tau = {{\left( {\rho c_{p} } \right)_{p} } \mathord{\left/ {\vphantom {{\left( {\rho c_{p} } \right)_{p} } {\left( {\rho c_{p} } \right)_{f} }}} \right. \kern-\nulldelimiterspace} {\left( {\rho c_{p} } \right)_{f} }}} \right)$ is ratio of nanoparticle heat capability to heat capability of fluid, $\sigma _{T}$ be the temperature surface tension coefficient, $\sigma _{\Phi }$ is the coefficient of concentration surface tension and $\left( {D_{T} } \right)$ is thermophoresis coefficient.

Following suitable similarity transformations are used for normalizing the system of PDE ^41^:

9 $$\left. \begin{gathered} \zeta = \sqrt {\frac{c}{\nu }} y,u = cxf^{\prime}\left( \zeta \right),v = - \sqrt {c\nu } f\left( \zeta \right), \hfill \\ \theta \left( \zeta \right) = \frac{{T - T_{\infty } }}{{T_{s} - T_{\infty } }},\phi \left( \zeta \right) = \frac{{\Phi - \Phi _{\infty } }}{{\Phi _{s} - \Phi _{\infty } }},\chi \left( \zeta \right) = \frac{{N - N_{\infty } }}{{N_{s} - N_{\infty } }}. \hfill \\ \end{gathered} \right\},$$

The reduced system will:

10 $$\begin{gathered} f^{{\prime \prime \prime }} - f^{{\prime 2}} + ff^{{\prime \prime }} + \beta _{1} \left( {2ff^{\prime } f^{{\prime \prime }} - f^{2} f^{{\prime \prime \prime }} } \right) + \beta _{2} \left( {f^{3} f^{{iv}} - 2ff^{{\prime 2}} f^{{\prime \prime }} - 3f^{2} f^{{\prime \prime 2}} } \right) \hfill \\ + \beta _{3} \left( {f^{{\prime \prime 2}} - ff^{{iv}} } \right) - M^{2} \left( {f^{\prime } - \beta _{1} ff^{{\prime \prime }} + \beta _{2} f^{2} f^{{\prime \prime \prime }} } \right) + \cos \alpha S\left( {\theta - Nr\phi - Nc\chi } \right) = 0, \hfill \\ \end{gathered}$$

Here $$\beta _{1} \left( { = c\lambda _{1} } \right),\beta _{2} \left( { = c^{2} \lambda _{2} } \right)\& \beta _{3} \left( { = c\lambda _{3} } \right)$$ are Deborah numbers, the buoyancy ratio parameter $$Nr\left( { = \frac{{\left( {\rho _{p} - \rho _{f} } \right)\left( {\Phi _{s} - \Phi _{\infty } } \right)}}{{\left( {1 - \Phi _{\infty } } \right)\left( {T_{\infty } } \right)\rho _{f} \beta ^{{**}} }}} \right)$$, $$M^{2} \left( { = \frac{{\sigma B_{0}^{2} }}{{\rho _{f} c}}} \right)$$ is the Hartman number, the mixed convection parameter is $$S\left( { = \frac{{\beta ^{{**}} g^{*} \left( {1 - \Phi _{\infty } } \right)\left( {T_{s} - T_{\infty } } \right)}}{{aU_{s} }}} \right)$$, the bioconvection Rayleigh number is $$Nc\left( { = \frac{{\gamma \left( {\rho _{m} - \rho _{f} } \right)\left( {N_{s} - N_{\infty } } \right)}}{{\left( {1 - \Phi _{\infty } } \right)\left( {T_{\infty } } \right)\rho _{f} \beta ^{{**}} }}} \right)$$.

11 $$\left( {1 + Rd\left( {1 + \left( {\theta _{w} - 1} \right)\theta ^{3} } \right)} \right)\theta ^{{\prime \prime }} + \Pr \left( {f\theta ^{\prime } - 2f^{\prime } \theta + Nb\phi ^{\prime } \theta ^{\prime } + Nt\theta ^{{\prime 2}} } \right) + Q_{T} \theta + Q_{E} \exp \left( { - n\zeta } \right) = 0,$$

Here $Nb\left( { = \frac{{\tau D_{B} \left( {\Phi _{s} - \Phi _{\infty } } \right)}}{{\alpha _{m} }}} \right)$ is the Brownian motion parameter, $Pr\left( { = \frac{\nu }{{\alpha _{m} }}} \right)$ is the Prandtl number, $Rd\left( { = \frac{{16\sigma ^{*} T_{\infty }^{3} }}{{3kk^{*} }}} \right)$ is the radiation parameter, $\theta _{w} \left( { = \frac{{T_{s} }}{{T_{\infty } }}} \right)$ is temperature ratio parameter, $Q_{T} \left( { = \frac{{Q_{T}^{*} }}{{\left( {\rho c_{p} } \right)_{f} a}}} \right)$ is temperature dependent heat source/sink parameter, $Q_{E} \left( { = \frac{{Q_{E}^{*} }}{{\left( {\rho c_{p} } \right)_{f} a}}} \right)$ be the exponential space-based heat source/sink parameter, $Nt\left( { = \frac{{\tau D_{T} \left( {T_{s} - T_{\infty } } \right)}}{{T_{\infty } \alpha _{m} }}} \right)$ is the thermophoresis parameter.

12 $$\phi ^{\prime\prime} + Le\Pr \left( {f\phi ^{\prime} - 2f^{\prime}\phi } \right) + \frac{{Nt}}{{Nb}}\theta ^{\prime\prime} - Le\Pr \sigma _{1} (1 + \delta \theta )^{m} exp\left( {\frac{{ - E}}{{1 + \delta \theta }}} \right)\phi = 0,$$

Here $Le\left( { = \frac{{\alpha _{m} }}{{D_{B} }}} \right)$ is Lewis's number, $\sigma _{1} = \frac{{Kr^{2} }}{c}$ be the chemical reaction parameter, $E = \frac{{E_{a} }}{{K_{1} T_{\infty } }}$ signifies the activation energy, $\delta = \frac{{T_{s} - T_{\infty } }}{{T_{\infty } }}$ clarify the temperature difference parameter.

13 $$\chi '' + Lbf\chi ' - Pe\left( {\phi ''\left( {\chi + \delta _{1} } \right) + \chi '\phi '} \right) = 0,$$

Here $Lb\left( { = \frac{\nu }{{D_{m} }}} \right)$ is bioconvection Lewis number, $\delta _{1} \left( { = \frac{{N_{\infty } }}{{N_{s} - N_{\infty } }}} \right)$ is microorganism difference parameter $Pe\left( { = \frac{{bW_{c} }}{{D_{m} }}} \right)$ is Peclet number.

With dimensionless boundary constraints:

14 $$\left. \begin{gathered} f\left( \zeta \right) = 0,f^{\prime}\left( \zeta \right) = - C\left( {1 + D} \right), \hfill \\ \theta ^{\prime}\left( \zeta \right) = - S_{1} \left( {1 - \theta \left( \zeta \right)} \right),\phi ^{\prime}\left( \zeta \right) = - S_{2} \left( {1 - \phi \left( \zeta \right)} \right), \hfill \\ \chi ^{\prime}\left( \zeta \right) = - S_{3} \left( {1 - \chi \left( \zeta \right)} \right),at\zeta = 0, \hfill \\ f^{\prime}\left( \zeta \right) \to 0,\theta \left( \zeta \right) \to 0,\phi \left( \zeta \right) \to 0,\chi \left( \zeta \right) \to 0,\,as\,\zeta \to \infty . \hfill \\ \end{gathered} \right\},$$

Here $C\left( { = \frac{{\sigma _{T} A}}{{\mu c}}\sqrt {\frac{\nu }{a}} } \right)$ is the Marangoni number, and $D\left( { = \frac{{\sigma _{\Phi } B}}{{\sigma _{T} A}}} \right)$ is Marangoni ratio parameter, $S_{1} \left( { = \frac{{h_{f} }}{k}\sqrt {\frac{\nu }{a}} } \right)$ is thermal Biot number, $S_{2} \left( { = \frac{{h_{g} }}{{D_{B} }}\sqrt {\frac{\nu }{a}} } \right)$ is concentration Biot number, $S_{3} \left( { = \frac{{h_{n} }}{{D_{m} }}\sqrt {\frac{\nu }{a}} } \right)$ is microorganism Biot number.

The shear stress of Burger fluid describes as^10^

15 $$\begin{gathered} \left( {1 + \lambda _{1} \frac{D}{{Dt}} + \lambda _{2} \frac{{D^{2} }}{{D_{{t^{2} }} }}} \right)S_{{xy}} = \mu \left( {\frac{{\partial u}}{{\partial y}} + \frac{{\partial v}}{{\partial x}}} \right) \hfill \\ + \mu \lambda _{3} \left( {u\frac{{\partial ^{2} u}}{{\partial x\partial y}} + v\frac{{\partial ^{2} u}}{{\partial y^{2} }} + u\frac{{\partial v}}{{\partial x^{2} }} + v\frac{{\partial ^{2} v}}{{\partial x\partial y}} - 2\frac{{\partial u}}{{\partial y}}\frac{{\partial v}}{{\partial x}} - \left( {\frac{{\partial u}}{{\partial y}}} \right)^{2} - \left( {\frac{{\partial v}}{{\partial x}}} \right)^{2} } \right) \hfill \\ \end{gathered}$$

Here above equation signifies that it is impossible to write shear stress in current case in terms of component of velocity $u,v$. It shows the way to detail that shear stress according in terms of *f* and derivative with respect to $\zeta$ by using transformation (9). According to this point of view, one cannot calculate skin friction in this situation because

16 $$C_{f} = \frac{{\left. {S_{{xy}} } \right|_{{y = 0}} }}{{\rho U_{w}^{2} }}.$$

In which skin friction for viscous fluid ($\lambda _{1} ,\lambda _{2} ,\lambda _{3} = 0$) is $f^{\prime\prime}\left( 0 \right)$.

The Nusselt number, Sherwood number, and microorganism’s density number can be described as:

17 $$Nu = \frac{{xq_{s} }}{{k\left( {T_{s} - T_{\infty } } \right)}},Sh = \frac{{xq_{w} }}{{D_{B} \left( {\Phi _{s} - \Phi _{\infty } } \right)}},Sn = \frac{{xq_{n} }}{{D_{m} \left( {N_{s} - N_{\infty } } \right)}},$$

18 $$q_{s} = - k\left( {T_{y} } \right)_{{y = 0}} ,q_{w} = - D_{B} \left( {\Phi _{y} } \right)_{{y = 0}} ,q_{n} = - D_{m} \left( {N_{y} } \right)_{{y = 0}} ,$$

Hence, the dimensionless form of engineering quantities is given by

19 $$\frac{{Nu}}{{\text{Re} _{x}^{{\frac{1}{2}}} }} = - \theta ^{\prime}\left( \zeta \right),\frac{{Sh}}{{\text{Re} _{x}^{{\frac{1}{2}}} }} = - \phi ^{\prime}\left( \zeta \right),\frac{{Sn}}{{\text{Re} _{x}^{{\frac{1}{2}}} }} = - \chi ^{\prime}\left( \zeta \right),$$

## Numerical approach

The two-dimensional nanofluid movement of Burgers fluid over the inclined wall is discussed in this section. Momentum, temperature, the concentration of nanomaterials, and swimming motile microorganism Eqs. (10–14) with relevant convective boundary conditions (14) are converted to a stronger non-dimensional system of ordinary differential equations using similarity transformations. Various values of several parameters are resolved numerically using the MATLAB computational toot inherent in bvp4c. This bvp4c process is used for three Lobatto-IIIa formulas. This formula is used for collective numerical results. Introduce the following new variables expressed as:

Let,

20 $$\left. \begin{gathered} f = p_{1} ,f^{\prime} = p_{2} ,f^{\prime\prime} = p_{3} ,f^{\prime\prime\prime} = p_{4} ,f^{{iv}} = p^{\prime}_{4} , \hfill \\ \theta = p_{5} ,\theta ^{\prime} = p_{6} ,\theta ^{\prime\prime} = p^{\prime}_{6} , \hfill \\ \phi = p_{7} ,\phi ^{\prime} = p_{8} ,\phi ^{\prime\prime} = p^{\prime}_{8} , \hfill \\ \chi = p_{9} ,\chi ^{\prime} = p_{{10}} ,\chi ^{\prime\prime} = p^{\prime}_{{10}} , \hfill \\ \end{gathered} \right\},$$

21 $$p^{\prime}_{4} = \frac{\begin{gathered} - p_{4} + p_{2} ^{2} - p_{1} p_{3} - \beta _{1} \left( {2p_{1} p_{2} p_{3} - p_{1} ^{2} p_{4} } \right) - \beta _{2} \left( { - 2p_{1} p_{2} ^{2} p_{3} - 3p_{1} ^{2} p_{3} ^{2} } \right) \hfill \\ - \beta _{3} \left( {p_{3} ^{2} } \right) + M^{2} \left( {p_{2} - \beta _{1} p_{1} p_{3} + \beta _{2} p_{1} ^{2} p_{4} } \right) - \cos \alpha S\left( {p_{5} - Nrp_{7} - Ncp_{9} } \right)\, \hfill \\ \end{gathered} }{{\left( {\beta _{2} p_{1} ^{3} - \beta _{3} p_{1} } \right)}},$$

22 $$p^{\prime}_{6} = \frac{{ - \Pr \left( {p_{1} p_{6} - 2p_{2} p_{5} + Nbp_{8} p_{6} + Ntp_{6} ^{2} } \right) - Q_{T} p_{5} - Q_{E} \exp \left( { - n\zeta } \right)}}{{\left( {1 + Rd\left( {1 + \left( {\theta _{w} - 1} \right)p_{5}^{3} } \right)} \right)}},$$

23 $$p^{\prime}_{8} = - Le\Pr \left( {p_{1} p_{8} - 2p_{2} p_{7} } \right) - \frac{{Nt}}{{Nb}}p^{\prime}_{6} + Le\Pr \sigma _{1} (1 + \delta p_{5} )^{m} exp\left( {\frac{{ - E}}{{1 + \delta p_{5} }}} \right)p_{7} ,$$

23 $$p^{\prime}_{{10}} = - Lbp_{1} p_{{10}} + Pe\left( {p^{\prime}_{8} \left( {p_{9} + \delta _{1} } \right) + p_{{10}} p_{8} } \right),$$

With

24 $$\left. \begin{gathered} p_{1} \left( \zeta \right) = 0,\left. {p_{2} \left( \zeta \right)} \right| = - C\left( {1 + D} \right), \hfill \\ p_{6} \left( \zeta \right) = - S_{1} \left( {1 - p_{5} \left( \zeta \right)} \right),p_{8} \left( \zeta \right) = - S_{2} \left( {1 - p_{7} \left( \zeta \right)} \right), \hfill \\ p_{{10}} \left( \zeta \right) = - S_{3} \left( {1 - p_{9} \left( \zeta \right)} \right),at\zeta = 0, \hfill \\ p_{2} \left( \zeta \right) \to 0,p_{5} \left( \zeta \right) \to 0,p_{7} \left( \zeta \right) \to 0,p_{9} \left( \zeta \right) \to 0,\,as\,\zeta \to \infty . \hfill \\ \end{gathered} \right\},$$

## Results and discussion

**Figure 2:**
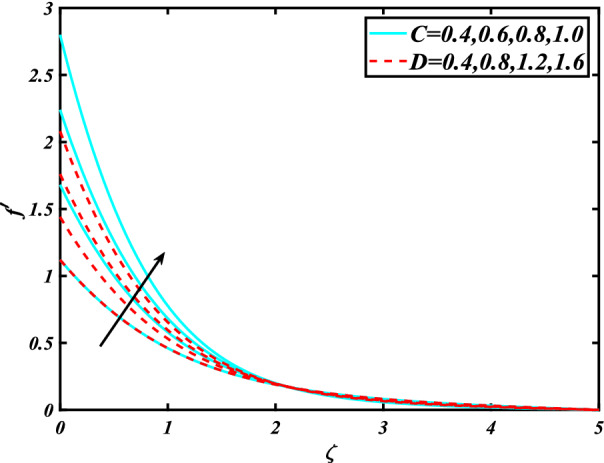
Significance of $C\& D$ for $f^{\prime}$.

**Figure 3:**
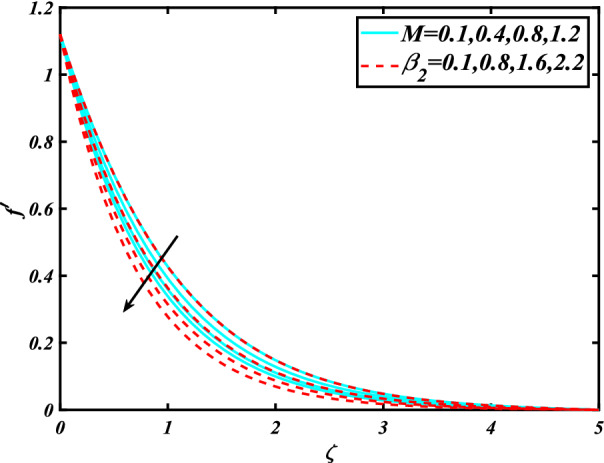
Significance of $M\& \beta _{2}$ for $f^{\prime}$.

**Figure 4:**
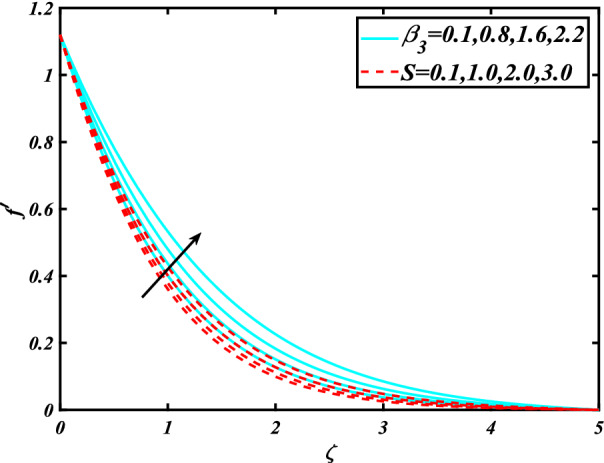
Significance of $\beta _{3} \& S$ for $f^{\prime}$.

**Figure 5:**
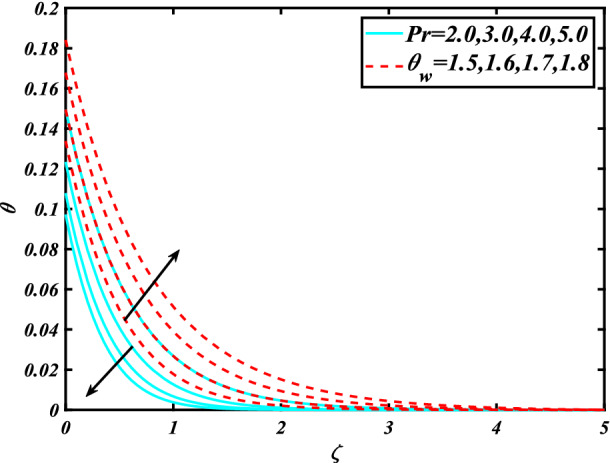
Significance of $Pr\& \theta _{w}$ for $\theta$.

**Figure 6:**
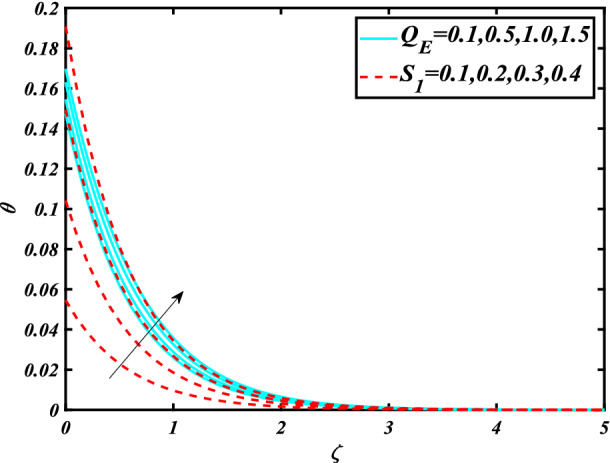
Significance of $Q_{E} \& S_{1}$ for $\theta$.

**Figure 7:**
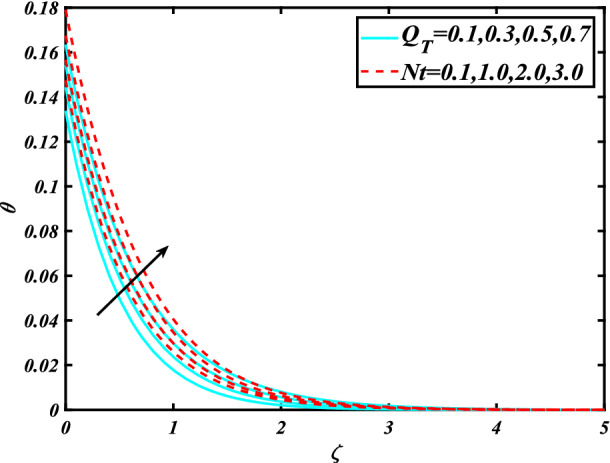
Significance of $Q_{T} \& Nt$ for $\theta$.

**Figure 8:**
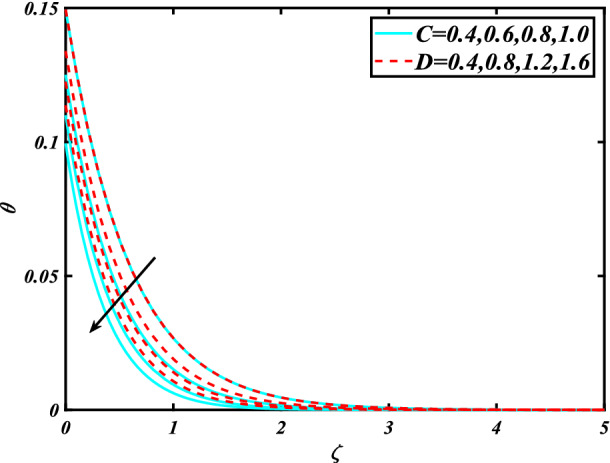
Significance of $C\& D$ for $\theta$.

**Figure 9:**
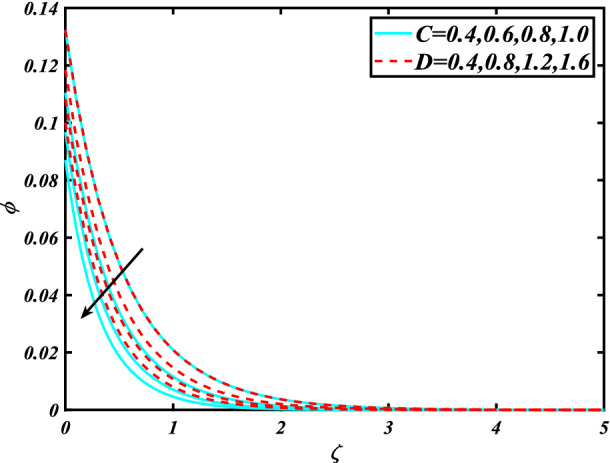
Significance of $C\& D$ for $\phi$.

**Figure 10:**
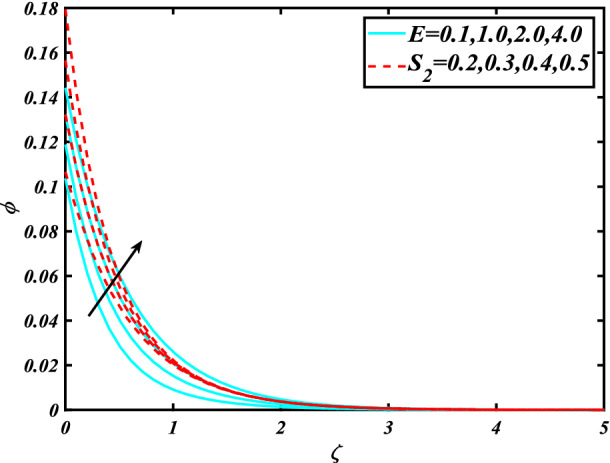
Significance of $E\& S_{2}$ for $\phi$.

**Figure 11:**
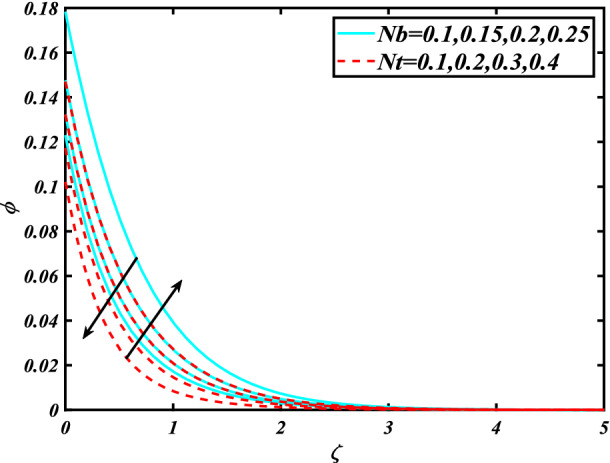
Significance of $Nb\& Nt$ for $\phi$.

**Figure 12:**
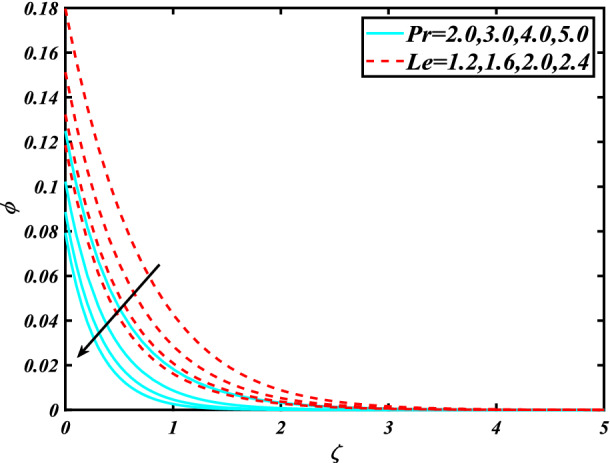
Significance of $\Pr \& Le$ for $\phi$.

**Figure 13:**
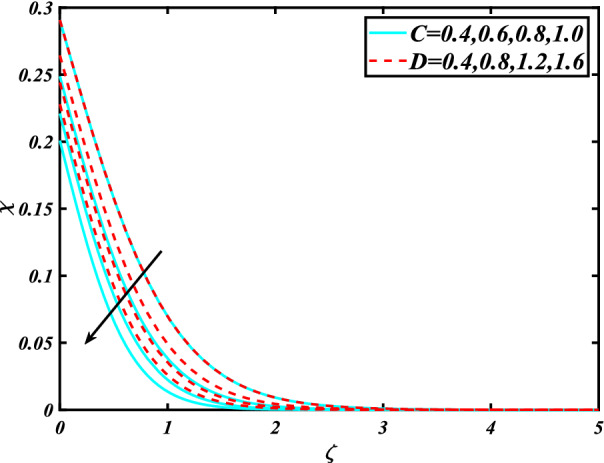
Significance of $C\& D$ for $\chi$.

**Figure 14:**
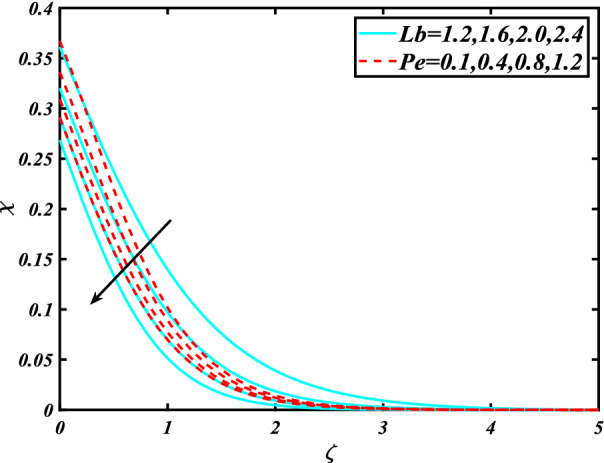
Significance of $Lb\,\& \,Pe$ for $\chi$.

In this section, the physical behavior of various parameters (buoyancy ratio parameter, Hartman number, mixed convection parameter, thermophoresis parameter, Brownian motion parameter, Prandtl number, temperature ratio parameter, thermal dependent heat source/sink parameter, exponential space dependent heat source/sink parameter, Lewis's number, bioconvection Lewis number, bioconvection Rayleigh number, Peclet number, Marangoni number, Marangoni ratio parameter, thermal Biot number, concentration Biot number, and microorganism Biot number ) against subjective flow fields are discussed in detail and depicted through Fig. 2, 3, 4, 5, 6, 7, 8, 9, 10, 11, 12, 13 and 14. Figure 2 is designed to notice the trends of velocity field $f^{\prime}$ with exaggerate of distinguishing Marangoni number $C$ and Marangoni ratio parameter $D$. It is analyzed that the higher values of the Marangoni number $C$ and Marangoni ratio parameter $D$ provide a enhancing trend in the velocity of the fluid $f^{\prime}$. Figure 3 reveals the behavior of the Hartman number $M$ and $\beta _{2}$ on the flow of rate type nanomaterials $f^{\prime}$. Velocity $f^{\prime}$ curves preserve reducing phenomenon for greater Hartman number $M$ and $\beta _{2}$. Variation of velocity field $f^{\prime}$ with mixed convection parameter $S$ and $\beta _{3}$ is captured in Fig. 4. It is seen that velocity of rate type nanoliquid rises for larger magnitudes of $S$ also depicted that velocity is increased for higher estimation of $\beta _{3}$. The physical explanation referred to like enhancing trend is justified as mixed convection parameter currents the ratio between buoyancy force to viscous force. The outcomes of temperature distribution $\theta$ against temperature ratio parameter $\theta _{w}$ and Prandtl number $\Pr$ are validated in Fig. 5. The temperature distribution $\theta$ rises by the uprising temperature ratio parameter $\theta _{w}$ while dwindles for a higher amount of Prandtl number $\Pr$. The estimation in temperature distribution $\theta$ concerning thermal Biot number $S_{1}$ and exponential space dependent source/sink parameter $Q_{E}$ is displayed in Fig. 6. From the curves of thermal Biot number $S_{1}$ and exponential space dependent source/sink parameter $Q_{E}$, it is observed that enhance in thermal Biot number $S_{1}$ and exponential space dependent source/sink parameter $Q_{E}$ enhances temperature distribution $\theta$. Figure 7 examined features of the thermophoresis parameter $Nt$ and thermal dependent source/sink parameter $Q_{T}$ for temperature distribution $\theta$. One can depict from this figure thermal field $\theta$ is increase with a higher amount of both the physical parameter thermophoresis parameter $Nt$ and thermal dependent source/sink parameter $Q_{T}$.From physical point of view, we can say that an upsurge in the strength of thermophoresis affects an effective movement of the nanomaterials which improves the thermal conductivity of the fluid which outcomes into augmentation of the fluid temperature. Figure 8 is captured to illustrate the behavior of the Marangoni number $C$ and Marangoni ratio parameter $D$ against a thermal field of species $\theta$. It is scrutinized that the thermal field of species $\theta$ is declined for higher estimation of Marangoni number $C$ and Marangoni ratio parameter $D$. The impression of the Marangoni number $C$ and Marangoni ratio parameter $D$ on the volumetric concentration of nanoparticles $\phi$ is demonstrated in Fig. 9. The reduction in the concentration field $\phi$ is scrutinized by growing the magnitude of the Marangoni number $C$ and Marangoni ratio parameter $D$. Figure 10 illustrates the impact of activation energy parameter $E$ and concentration Biot number $S_{2}$ on the concentration of nanoparticles $\phi$. It is analyzed that the concentration of species $\phi$ boosted up with larger activation energy parameter $E$ and concentration Biot number $S_{2}$.Fig. 11 is captured to scrutinize the behavior $Nt$ and Brownian motion parameter $Nb$ against the rescaled density of the concentration profile $\phi$. The concentration profile $\phi$ upsurges for thermophoresis parameter $Nt$ while reducing for Brownian motion parameter $Nb$. Physically when we increase the thermophoresis and Brownian motion, the thermal efficiency of fluid rises. From this scenario noticed that the thermophoresis is also increased which tends to move nanoparticles from warm to cold sections. Features of concentration profile $\phi$ over the Prandtl number $\Pr$ and Lewis's number $Le$ for concentration are plotted in Fig. 12. From the curves of the concentration profile declines for the Larger Prandtl number $\Pr$. Physically, Prandtl number illustrates ratio between momentum diffusivity to thermal diffusivity. Furthermore, Lewis's number $Le$ causes a reduction in the volumetric concentration nanoparticle field $\phi$. Figure 13 is prepared to estimate the trends of Marangoni number $C$ and Marangoni ratio parameter $D$ against the concentration of microorganism $\chi$. Here the concentration of microorganism $\chi$ depressed with a larger estimation of Marangoni number $C$ and Marangoni ratio parameter $D$. The salient characteristics of Peclet number $Pe$ and $Lb$ against microorganism concentration $\chi$ are examined through Fig. 14. The microorganism’s profile $\chi$ decline by an increment in the estimation of both parameter Peclet number $Pe$ and bioconvection Lewis number $Lb$. Physically the microorganism’s density of motile microorganisms always is reduced due to a higher estimation of the Peclet number.

In this slice, the numerical outcomes of versus parameters via $- f^{\prime\prime}\left( 0 \right)$,$- \theta ^{\prime}\left( 0 \right)$,$- \phi ^{\prime}\left( 0 \right)$ and $- \chi ^{\prime}\left( 0 \right)$ are examined in Tables 1, 2, 3 and 4. Table 1 is calculated to investigate the trend of local skin friction coefficient $- f^{\prime\prime}\left( 0 \right)$ via flow parameters. The local skin friction coefficient $- f^{\prime\prime}\left( 0 \right)$ increased via $C$ and $D$ while decline for $\lambda$. Table 2 is explored to scrutinize the aspects of local Nusselt number $- \theta ^{\prime}\left( 0 \right)$ for flow parameters. From mathematical data investigation, it is examined that local Nusselt number $- \theta ^{\prime}\left( 0 \right)$ reduces with the improvement of $Nb$. Table 3 reveals the variation of local Sherwood number $\phi ^{\prime}\left( 0 \right)$ via greater estimations of different parameters. From this table disclosed that local Sherwood number $- \phi ^{\prime}\left( 0 \right)$ rises for $Pr\& S_{2}$. The numerical outcomes of local microorganism numbers $- \chi ^{\prime}\left( 0 \right)$ via flow parameters are shown in Table 4. Now local density number of $- \chi ^{\prime}\left( 0 \right)$ enhanced for higher variations $C\& Lb$. Table 5 presents a comparative work of the current outcomes with refs. ^8,43^. Here good agreement is observed with current results and published literature Ref. ^8,43^.

**Table 1:** Outcomes of $- f^{\prime\prime}\left( 0 \right)$ versus flow parameter.

Flow parameters						Local skin friction coefficients
$\lambda$	$M$	$Nr$	$Nc$	$C$	$D$	$- f^{\prime\prime}\left( 0 \right)$
0.1 0.6 1.2	0.1	0.5	0.5	0.4	0.4	1.0172 1.0167 1.0057
0.2	0.2 0.5 0.7	0.5	0.5	0.4	0.4	1.0255 1.0942 1.1880
0.2	0.1	0.2 1.0 2.0	0.5	0.4	0.4	1.0066 1.0275 1.0292
0.2	0.1	0.5	0.2 1.0 2.0	0.4	0.4	1.9265 1.0281 1.3283
0.2	0.1	0.5	0.5	0.5 0.8 1.1	0.4	1.3532 1.4843 3.6851
0.2	0.1	0.5	0.5	0.4	0.5 1.0 1.5	1.1039 1.6118 2.1507

**Table 2:** Outcomes of $- \theta ^{\prime}\left( 0 \right)$ versus flow parameters.

Flow parameters								Local Nusselt number
$\Pr$	$Nb$	$Nt$	$Le$	$S_{1}$	$C$	$D$	$\theta _{w}$	$- \theta ^{\prime}\left( 0 \right)$
3.0 5.0 7.0	0.2	0.3	2.0	0.5	0.5	0.3	1.5	0.4121 0.4212 0.4314
2.0	0.1 0.6 1.2	0.3	2.0	0.5	0.5	0.3	1.5	0.3844 0.3800 0.3746
2.0	0.2	0.1 0.6 1.2	2.0	0.5	0.5	0.3	1.5	0.3856 0.3804 0.3738
2.0	0.2	0.3	1.2 3.0 5.0	0.5	0.5	0.3	1.5	0.3834 0.3836 0.3839
2.0	0.2	0.5	2.0	0.1 0.8 1.6	0.5	0.3	1.5	0.0944 0.5358 0.7978
2.0	0.2	0.3	2.0	0.5	0.1 1.0 2.0	0.3	1.5	0.3986 0.4121 0.4227
2.0	0.2	0.3	2.0	0.5	0.5	0.1 0.4 0.7	1.5	0.3867 0.3991 0.4079
2.0	0.2	0.3	2.0	0.5	0.5	0.3	1.6 1.7 1.8	0.3936 0.3709 0.3500

**Table 3:** Outcomes of $- \phi ^{\prime}\left( 0 \right)$ versus flow parameters.

Flow parameters							Local Sherwood number
$\Pr$	$Nb$	$Nt$	$Le$	$S_{2}$	$C$	$D$	$- \phi ^{\prime}\left( 0 \right)$
3.0 5.0 7.0	0.2	0.3	2.0	0.5	0.5	0.3	0.4163 0.4344 0.4443
2.0	0.1 0.6 1.2	0.3	2.0	0.5	0.5	0.3	0.3659 0.4211 0.4266
2.0	0.2	0.1 0.6 1.2	2.0	0.5	0.5	0.3	0.4204 0.3685 0.3131
2.0	0.2	0.3	1.2 3.0 5.0	0.5	0.5	0.3	0.3650 0.4149 0.4402
2.0	0.2	0.5	2.0	0.1 0.8 1.6	0.5	0.3	0.0892 0.4639 0.7896
2.0	0.2	0.3	2.0	0.5	0.1 1.0 2.0	0.3	0.4079 0.4248 0.4328
2.0	0.2	0.3	2.0	0.5	0.5	0.1 0.4 0.7	0.4018 0.4130 0.4209

**Table 4:** Outcomes of $- \chi ^{\prime}\left( 0 \right)$ versus flow parameters.

Flow parameters					Local Microorganism number
$S_{3}$	$Lb$	$Pe$	$C$	$D$	$- \chi ^{\prime}\left( 0 \right)$
0.1 0.6 1.2	2.0	0.1	0.4	0.4	0.0911 0.3774 0.5445
0.5	1.2 1.8 2.6	0.1	0.4	0.4	0.2624 0.2927 0.3087
0.5	1.0	0.2 0.8 1.6	0.4	0.4	0.2778 0.3139 0.3341
0.5	1.0	0.1	0.5 0.8 1.1	0.4	0.4392 0.3739 0.3892
0.5	1.0	0.1	0.4	0.5 1.0 1.5	0.3408 0.3564 0.3681

**Table 5:** Comparison of obtained outcomes in limiting case when $S = Nr = Nc = 0 = Q_{T} = Q_{E}$ and $Pe = Lb = 0$.

$\Pr$	Rashidi et al.^43^	Rashidi et al.^8^	Our results
1.0	−1.710937	−1.710936	−1.710936
2.0	−2.458997	−2.486000	−2.486000
3.0	−3.028177	−3.028170	−3.028170
4.0	−3.585192	−3.585189	−3.585189
5.0	−4.028540	−4.028533	−4.028533

## Conclusion

The current article discloses the impact of activation energy in the bioconvective flow of Burger nanofluid by an inclined wall. The heat transfer mechanism of Burger nanofluid is analyzed through the nonlinear thermal radiation effect. The Brownian dispersion and thermophoresis diffusions aspects are also scrutinized. The behavior of distinguishing crucial parameters is scrutinized on the flow of fluid, thermal field, solutal field, and microorganism’s field. The main outcomes are worth mentioning:

- The velocity profile declined for the greater magnitude of magnetic parameter.
- The velocity profile improved for the values of mixed convection parameter.
- The temperature profile increases for temperature dependent heat source/sink parameter and exponential space-based heat source/sink parameter while declining with Prandtl number, and Marangoni ratio parameter
- The concentration of species profile boosts for thermophoresis parameter and activation energy
- The concentration profile diminishes for higher values of $Le$ while enlarging with mass Biot number
- The concentration of microorganisms is decreased by enhancing the variation of the Marangoni number and Marangoni ratio parameter
- The microorganism field depressed by enhancing the values of the Peclet number.

## Abbreviations

$\left( {u\& v} \right)$: Velocity components, ms^−^^1^
$$\left( {\alpha _{m} } \right)$$: Thermal diffusivity
$\left( \alpha \right)$: Inclination of the wall
$\gamma$: Microorganisms average volume, m^3^
$\left( {\rho _{f} } \right)$: Density of fluid, kgm^−^^3^
$W_{c}$: Cell Swimming Speed, ms^−^^1^
$\left( {\rho c_{p} } \right)_{p}$: Heat Capacitance of nanoparticles, Jm^−^^3^ K^−^^1^
$\left( {\rho c_{p} } \right)_{f}$: Heat Capacitance of fluid, Jm^−^^3^ K^−^^1^
$\rho _{m}$: Density of Motile Microorganisms, kgm^−^^3^
$\left( {g^{*} } \right)$: Acceleration due to gravity
$$\left( \sigma \right)$$: Electrical conductivity
$\sigma _{1}$: Chemical reaction parameter
$$\beta ^{{**}}$$: Thermal suspension coefficient, K^−^^1^
$$\left( T \right)$$: Temperature *K*
$\left( \Phi \right)$: Concentration of nanoparticles, molL^−^^1^
$$\left( N \right)$$: Motile microorganisms, m^−^^3^
$\left( {D_{B} } \right)$: Brownian motion coefficient, m^2^s^−^^1^
$\left( {D_{T} } \right)$: Thermophoresis diffusion coefficient, m^2^s^−^^1^
$$D_{m}$$: Microorganisms coefficient, m^2^s^−^^1^
$$\left( {\lambda _{1} \& \lambda _{2} } \right)$$: Relaxation effects
$$\left( {\lambda _{3} } \right)$$: Retardation effect
$$\left( {\beta _{1} ,\beta _{2} \& \beta _{3} } \right)$$: Deborah numbers
$\left( {Nr} \right)$: Buoyancy ratio parameter
$$\left( {M^{2} } \right)$$: Hartman number
$\left( S \right)$: Mixed convection parameter
$Kr^{2}$: Chemical reaction constant, s^−^^1^
$\left( {Nc} \right)$: Bioconvection Rayleigh number
$h_{f}$: Convective heat transfer coefficient, Wm^2^K^−^^1^
$h_{g}$: Concentration transfer coefficient, ms^−^^1^
$h_{n}$: Microorganisms transfer coefficient, ms^−^^1^
$\left( {Nb} \right)$: Brownian motion parameter
$$\left( {Pr} \right)$$: Prandtl number
$$\left( {Rd} \right)$$: Radiation parameter
$\left( {\theta _{w} } \right)$: Temperature ratio parameter
$\left( {Q^{*} _{T} } \right)$: Thermal dependent heat source coefficient
$\left( {Q^{*} _{E} } \right)$: Exponential space dependent heat source
$\left( {Q_{E} } \right)$: Exponential space bases source parameter
$\left( {Q_{T} } \right)$: Temperature dependent Heat source parameter
$\left( {Q_{E} } \right)$: Exponential space bases source parameter
$$\left( {Nt} \right)$$: Thermophoresis parameter
$$\left( {Le} \right)$$: Lewis's number
$\left( {Lb} \right)$: Bioconvection Lewis number
$\left( {\delta _{1} } \right)$: Microorganism difference parameter
$$\left( {Pe} \right)$$: Peclet number
$$\left( C \right)$$: Marangoni number
$\left( D \right)$: Marangoni ratio parameter
$\left( {S_{1} } \right)$: Thermal Biot number
$\left( {S_{2} } \right)$: Concentration Biot number
$\left( {S_{3} } \right)$: Microorganism Biot number
$$\sigma _{q}$$: Boltzmann constant
$k^{*}$: Means absorption coefficient
$\sigma _{T}$: Temperature surface tension coefficient
$\sigma _{\Phi }$: Coefficient of concentration surface tension
$$q_{s}$$: Heat flux
$q_{n}$: Motile density flux
$$q_{w}$$: Mass flux
$$\left( {Nu} \right)$$: Nusselt number
$$\left( {Sh} \right)$$: Sherwood number
$\left( {Sn} \right)$: Microorganism’s density number
